# Targeting of miR-96-5p by catalpol ameliorates oxidative stress and hepatic steatosis in LDLr-/- mice via p66shc/cytochrome C cascade

**DOI:** 10.18632/aging.102721

**Published:** 2020-02-05

**Authors:** Yukun Zhang, Changyuan Wang, Jiawei Lu, Yue Jin, Canyao Xu, Qiang Meng, Qi Liu, Deshi Dong, Xiaodong Ma, Kexin Liu, Huijun Sun

**Affiliations:** 1 Department of Clinical Pharmacology, College of Pharmacy, Dalian Medical University, Dalian, China

**Keywords:** microRNA, catalpol, NAFLD, oxidative stress, hepatic steatosis

## Abstract

Hepatic steatosis and oxidative stress are considered to be the sequential steps in the development of non-alcoholic fatty liver disease (NAFLD). We previously found that catalpol, an iridoid glucoside extracted from the root of Romania glutinosa L, protected against diabetes-induced hepatic oxidative stress. Here, we found that the increased expression of p66shc was observed in NAFLD models and catalpol could inhibit p66shc expression to ameliorate NAFLD effectively. However, the underlying mechanisms remained unknown. The aim of the present study was to investigate the p66shc-targeting miRNAs in regulating oxidative stress and hepatic steatosis, also the mechanisms of catalpol inhibiting NAFLD. We found that the effects of catalpol inhibiting hepatic oxidative stress and steasis are dependent on inhibiting P66Shc expression. In addition, miR-96-5p was able to suppress p66shc/cytochrome C cascade via targeting p66shc mRNA 3’UTR, and catalpol could lead to suppression of NAFLD via upregulating miR-96-5p level. Thus, catalpol was effective in ameliorating NAFLD, and miR-96-5p/p66shc/cytochrome C cascade might be a potential target.

## INTRODUCTION

Hepatic steatosis and oxidative stress are considered to be the sequential steps in the development of non-alcoholic fatty liver disease (NAFLD) [1], which is becoming a new health challenge with the prevalence of 20% in general population. NAFLD encompasses the diseases from simple steatosis to steatohepatitis (NASH), fibrosis and cirrhosis. NAFLD also can cause hepatocellular carcinoma, liver failure and ultimately lead to premature death [2]. However, the mechanisms responsible for NAFLD have not been fully elucidated.

Oxidative stress is a condition due to an altered balance between the production of reactive oxygen species (ROS) and the antioxidant defenses capacity. It is recognized that oxidative stress involves complex cellular signaling. As indicated in previous study, NAFLD is associated with decreased oxygen consumption and adenosine triphosphate (ATP) generation, reduced total mitochondrial deoxyribonucleic acid (mtDNA), mitochondrial dysfunction and apoptosis [3], which all can be induced by oxidative stress. P66Shc is a ubiquitously expressed vertebrate protein, encoded by the human and mouse ShcA locus. It sustains the intracellular concentration of ROS by catalyzing their formation from the mitochondrial respiratory chain, triggering plasma membrane oxidases and suppressing ROS scavenging [4, 5]. Importantly, reports have indicated that high-fat diet and palmitate (PA) can increase p66Shc levels or activate it by stimulating Ser^36^ phosphorylation and share the ability to increase intracellular oxidative stress [6, 7]. Electron transfer between cytochrome C and p66shc is the key step to generate ROS and mitochondrial apoptosis [8]. Cytochrome C is regarded as the starting marker of apoptosis. The release of cytochrome C occurs prior to the activation of caspase and DNA fragmentation. In alcohol-induced liver apoptosis [9], nonalcoholic steatohepatitis [10], atherosclerosis [11] and most of metabolic and aging-related diseases, high levels of cytochrome C were detected. Although p66shc/cytochrome C pathway activation was observed during multiple aging-related diseases, its specific roles, as well as detailed mechanisms by which p66shc can be suppressed, require further investigation.

In recent years, research on NAFLD modulation has progressed, and several microRNAs have been shown involved in the pathophysiological processes of NAFLD. MiRNAs are short, endogenous, noncoding RNAs that have been considered as transcriptional or post-transcriptional regulators of gene expression. Mounts of studies have indicated important roles of microRNA mediated mechanisms in NAFLD [12, 13]. Despite these observations, whether a miRNA is involved in the fundamental pathogenesis of NAFLD, such as ROS accumulation, mitochondrial alteration and apoptosis through a p66shc related pathway remains unknown. For the reason that P66Shc is able to be regulated by miRNAs and based on miRNA target prediction programs miRanda (www.microrna.org/), TargetScan (www.targetscan.org/), and PicTar (http://pictar.mdc-berlin.de/), we hypothesized that a miRNA-P66Shc axis might be involved in regulating ROS accumulation, mitochondrial alteration and apoptosis, thus affecting NAFLD.

Catalpol is an iridoid glucoside and has been found to be present in large quantities in the root of Romania glutinosa L [14], whose structure was shown in Supplementary Figure 1. Catalpol has been demonstrated a variety of biological activities including anticancer, neuro-protective, anti-inflammatory, diuretic, hypoglycemic and anti-hepatitis virus effects in previous studies [15, 16]. Moreover, effects of catalpol on increasing mitochondrial biogenesis, enhancing endogenous antioxidant enzymatic activities and inhibiting free radical generation [17, 18] have also been reported. Based on the high potential of catalpol on inhibiting oxidative stress, we hypothesized that catalpol might ameliorate NAFLD via a p66shc related pathway by targeting miRNA.

Thus, the present study aimed to investigate the protective effects of catalpol on oxidative stress, apoptosis and hepatic steatosis, and to determine the role of miR-96-5p mediated p66shc/cytochrome C cascade in the protective effects of catalpol in mice and in vitro NAFLD models.

## RESULTS

### Catalpol ameliorated hepatic steatosis

**Figure 1:**
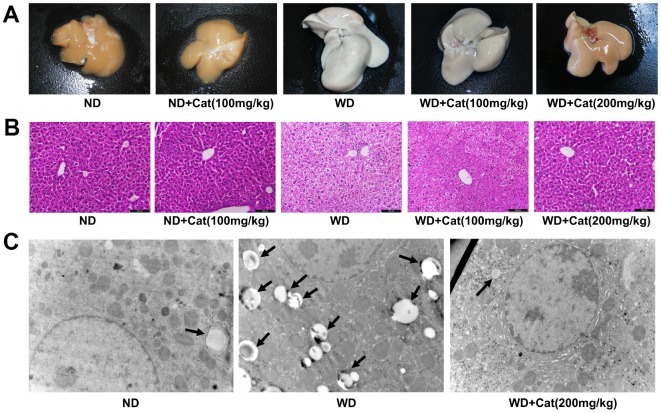
**Catalpol ameliorated hepatic steatosis in LDLr-/- mice.** The mice were fed with normal or western diet for 16-week, and then sacrificed for morphological and pathological observation. (**A**) Representative morphological images of liver sections. (**B**) H&E- stained sections of liver sections. H&E-stained sections were photographed at 200× magnification. (**C**) Transmission electron microscopy of liver sections. The liver sections were photographed at 10 000× magnification. The lipid droplets were indicated by arrows. ND: normal diet; WD: western diet.

At first, whether catalpol was able to protect against HFD-induced NAFLD in LDLr-/- mice was investigated by liver morphologic and histological examination. As shown in Figure 1A and 1B, ND-treated mice exhibited no apparent abnormalities, whereas long-term HFD feeding significantly increased the liver size, changed the color of the liver and induced a large amount of lipid deposition in hepatocytes, as well as massive nuclear pleomorphism and inflammatory cell infiltration. However, catalpol (200mg/kg) treatment markedly alleviated the liver injury and attenuated the hepatic lipid accumulation caused by the HFD. As shown in Figure 1C, in agreement, the results of transmission electron microscopy demonstrated a pronounced increase of hepatic lipid droplets in HFD-treated mice. However, hepatic lipid droplets accumulation was significantly decreased in catalpol treatment group compared with HFD-fed group. In addition, catalpol produced inhibitory effect on hepatic steatosis also confirmed in oxLDL or PA-treated hepG2 cells (Supplementary Figure 2A, 2B). Taken together, these results indicated that catalpol confered protection against HFD-induced NAFLD.

### Catalpol attenuated HFD-induced liver injury and lipid accumulation in HFD-treated LDLr-/- mice

**Figure 2:**
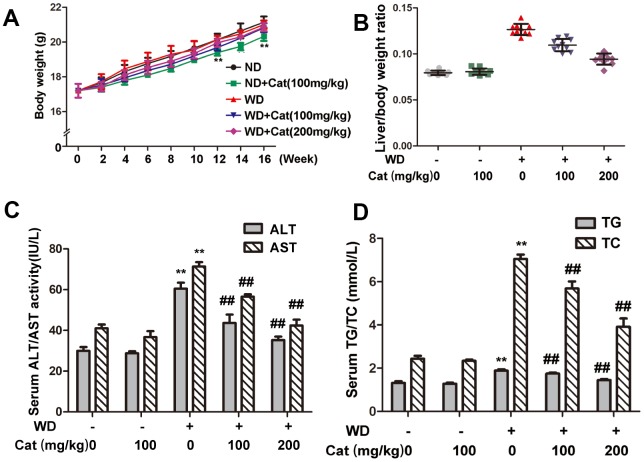
**Catalpol ameliorated liver index, hepatic injury in LDLr-/- mice.** (**A**) Body weights variation during 16 weeks period. (**B**) Liver index. (**C**) Serum levels of ALT and AST. (**D**) Serum levels of TC, TG. The results are the mean ± SD (n=10), ^**^P< 0.05 vs. Control group, ^##^P < 0.05 vs. WD group. ND: normal diet; WD: western diet.

We fortnightly measured body weights of mice, which were shown in Figure 2A. The body weight of mice in HFD-fed group didn’t change much compared with the body weight in ND-fed mice. However, catalpol decreased the body weight in ND-fed mice, without affecting food intake in mice (Supplementary Figure 3). Typical biochemical markers were employed to further elucidate the protective effects of catalpol against NAFLD. As shown in Figure 2B, the liver/body weight ratios in HFD-fed mice were markedly greater than those in the control group, and catalpol treatment obviously reversed this trend in a dose-dependent manner. Serum ALT and AST levels were also measured to elucidate the protective effect of catalpol. As shown in Figure 2C, compared with the control group, HFD-fed mice developed liver injury, as demonstrated by increased serum levels of ALT and AST. However, these changes were partly reversed by catalpol treatment. Plasma TG and TC were synthesized into the blood by the liver, thus plasma levels of TG and TC were detected to reflect liver function. As expected, after 16 weeks of HFD feeding, the serum levels of TG and TC were dramatically higher than those in the control group, whereas catalpol treatment partly abrogated these increases (Figure 2D). Above results suggested a protective effect of catalpol on HFD-induced hepatic injury.

### Catalpol ameliorated HFD-induced cell oxidative stress and cell apoptosis in HFD-treated LDLr-/- mice

**Figure 3:**
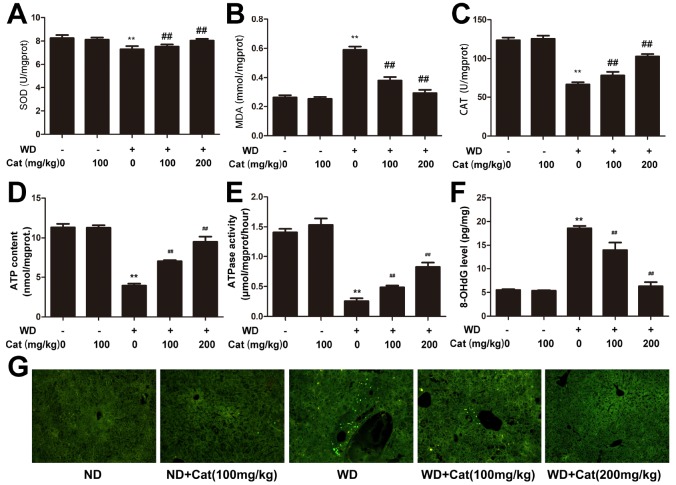
**Catalpol inhibited oxidative stress, promoted ATP production and inhibited hepatic apoptosis.** (**A**) SOD level. (**B**) MDA level. (**C**) CAT level. (**D**) ATP content. (**E**) ATPase activity. (**F**) 8-OHdG level. The results are the mean± SD (n=10), ^**^P< 0.05 vs. Control group, ^##^P < 0.05 vs. WD group. (**G**) Tunel staining for hepatic apoptosis. Tunel stained sections were photographed at 200× magnification. ND: normal diet; WD: western diet.

Lipid accumulation is closely link to oxidative stress and cellular apoptosis which may form a feedback loop that significantly aggravates NAFLD induced hepatic injury. We therefore evaluated the state of oxidative stress and cellular apoptosis. As shown in Figure 3A, compared with the control group, the HFD-fed group exhibited a significantly decreased SOD level. However, catalpol treatment effectively increased the SOD level. Additionally, catalpol could also decrease the MDA level increased by HFD-fed in LDLr-/- mice (Figure 3B). Moreover, compared with the control group, CAT level was significantly decreased in HFD-fed mice. In contrast, catalpol treatment markedly reversed the decreased CAT level (Figure 3C). Decreased ATP generation was also considered to be a result of oxidative stress and early progress to induce cell apoptosis. As shown in Figure 3D and 3E, ATP content and ATPase activity were significantly decreased in NAFLD mice compared with the control group (p<0.05). DNA oxidative damage level can be evaluated by 8-OHdG level. As shown in Figure 3F, 8-OHdG level was significantly increased in HFD-fed mice, and catalpol-treatment effectively reversed the increase induced by HFD. Catalpol-treatment resulted in increased ATP content and ATPase activity respectively. Further, hepatic cell apoptosis was detected by TUNEL staining. As shown in Figure 3G, compared with the control group, TUNEL positive cells which were stained in green were notably increased in HFD-fed mice. However, catalpol treatment significantly reduced the number of TUNEL positive cells. Additionally, effect of catalpol on inhibiting apoptosis was also detected in PA-treated hepG2 cells (Supplementary Figure 2C). Together, these results indicated that catalpol attenuated oxidative stress and apoptosis in NAFLD.

### Potential role of P66Shc in NAFLD

**Figure 4:**
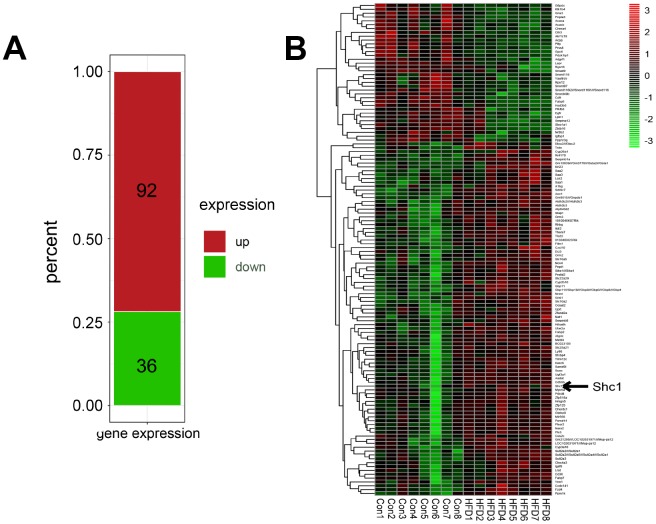
**Potential role of P66Shc in NAFLD.** (**A**) Differentially expressed genes retrieved from ‘GSE94754’ in the GEO dataset. (**B**) Heat map of differentially expressed genes retrieved from ‘GSE94754’ in the GEO dataset.

P66Shc expression was investigated in the high-fat diet and normal mice. Raw data were retrieved using the search terms ‘GSE94754’ in the GEO dataset. A total of128 different genes with p<0.05 and log F≥1.0 were identified. R_4.4 was used to screen the differentially expressed genes. The results indicated that 92 genes were significantly upregulated and 36 genes were remarkably decreased (Figure 4A). The analysis also indicated that p66shc expression in high-fat diet mice was significantly higher compared with the control group (Figure 4B). Together, the results suggested that p66shc might play an important role in the process of NAFLD.

### Protective effects of catalpol against NAFLD involved down-regulation of p66shc/cytochrome C pathway

**Figure 5:**
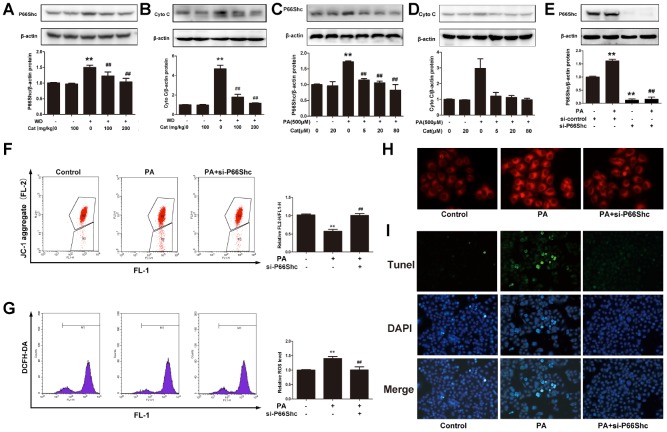
**P66shc was involved in catalpol-mediated protective effects.** (**A**) P66shc protein expression in NAFLD mice. (**B**) Cytochrome C (Cyto **C**) protein expression in NAFLD mice. (**C**) P66shc protein expression in PA- treated hepG2 cells. (**D**) Cyto C protein expression in PA-treated hepG2 cells. (**E**) P66shc protein expression was down-regulated by its specific siRNA. (**F**) JC-1 staining. (**G**) ROS level. (**H**) Nile red staining. (**I**) Tunel staining (Tunel positive cells were stained in green; DAPI was stained to show nuclei). The results are the mean± SD (n=10), ^**^P< 0.05 vs. Control group, ^##^P < 0.05 vs. WD or PA group.

In order to ensure whether p66shc/cytochrome C pathway was involved in NAFLD, p66shc and cytochrome C protein level was measured. As shown in Figure 5A, 5B, HFD-fed significantly increased p66shc and cytochrome C protein expression, however, catalpol reversed this increase in a dose-dependent manner. As expected, in PA or oxLDL-treated hepG2 cells, high expression of p66shc and cytochrome C level and the decrease after catalpol treatment were also observed (Figure 5C, 5D and supplementary Figure 4A, 4B). The results suggested a potential role of p66shc in NAFLD. Thus, p66shc expression was blocked by its specific siRNA for further investigation. In Figure 5E, p66shc protein expression was knocked down effectively. Then, the role of p66shc in ROS production, steatosis and apoptosis was measured. P66shc siRNA transfection significantly increased mitochondrial membrane potential (Figure 5F), inhibited ROS overproduction (Figure 5G), hepatic steatosis (Figure 5H) and cellular apoptosis (Figure 5I). These results indicated that decreased p66shc protein expression benefited to NAFLD through inhibiting oxidative stress, hepatic steatosis and these induced hepatic cell apoptosis.

### miR-96-5p was decreased among the miRNAs targeting p66shc in NAFLD

miRNAs are potent gene regulators that have been implicated in a wide range of diseases. Because NAFLD induced p66shc overexpression might be mediated by miRNAs, we investigated potential p66shc-regulating miRNAs. At First, the miRNA target prediction programs miRanda, TargetScan, and PicTar. were used to identify miRNAs that target the SIRT1 3′ untranslated region (3′UTR). The differential expression in the liver of the putative p66shc-targeting miRNAs was determined by real-time PCR with or without HFD As shown in Table 1, the levels of miR-9-5p and miR-124-3p in NAFLD mice were significantly increased to 2.69-fold and 3.04-fold compared with that in control mice. However, the level of miR-96-5p in NAFLD mice was decreased to 0.23-fold compared with that in control mice. In the current view, miRNAs can negatively regulate gene expression by binding with the 3′-UTR of a specific mRNA, causing its degradation or translational repression. Based on this mechanism, we hypothesized that the down-regulated miR-96-5p might play important roles in the regulation of NAFLD, possibly through inhibiting p66shc.

**Table 1:** Changes of the putative p66shc-targeting miRNAs in NAFLD.

**miRNA name**	**Fold change(NAFLD vs. control)**
miR-9-5p	2.69±0.61^**^
miR-96-5p	0.23±0.04^**^
miR-124-3p	3.04±0.40^**^

N=6,^**^p<0.05.

NAFLD, Nonalcoholic fatty liver disease; miRNA, microRNA.

### Increased miR-96-5p inhibits p66shc expression

**Figure 6:**
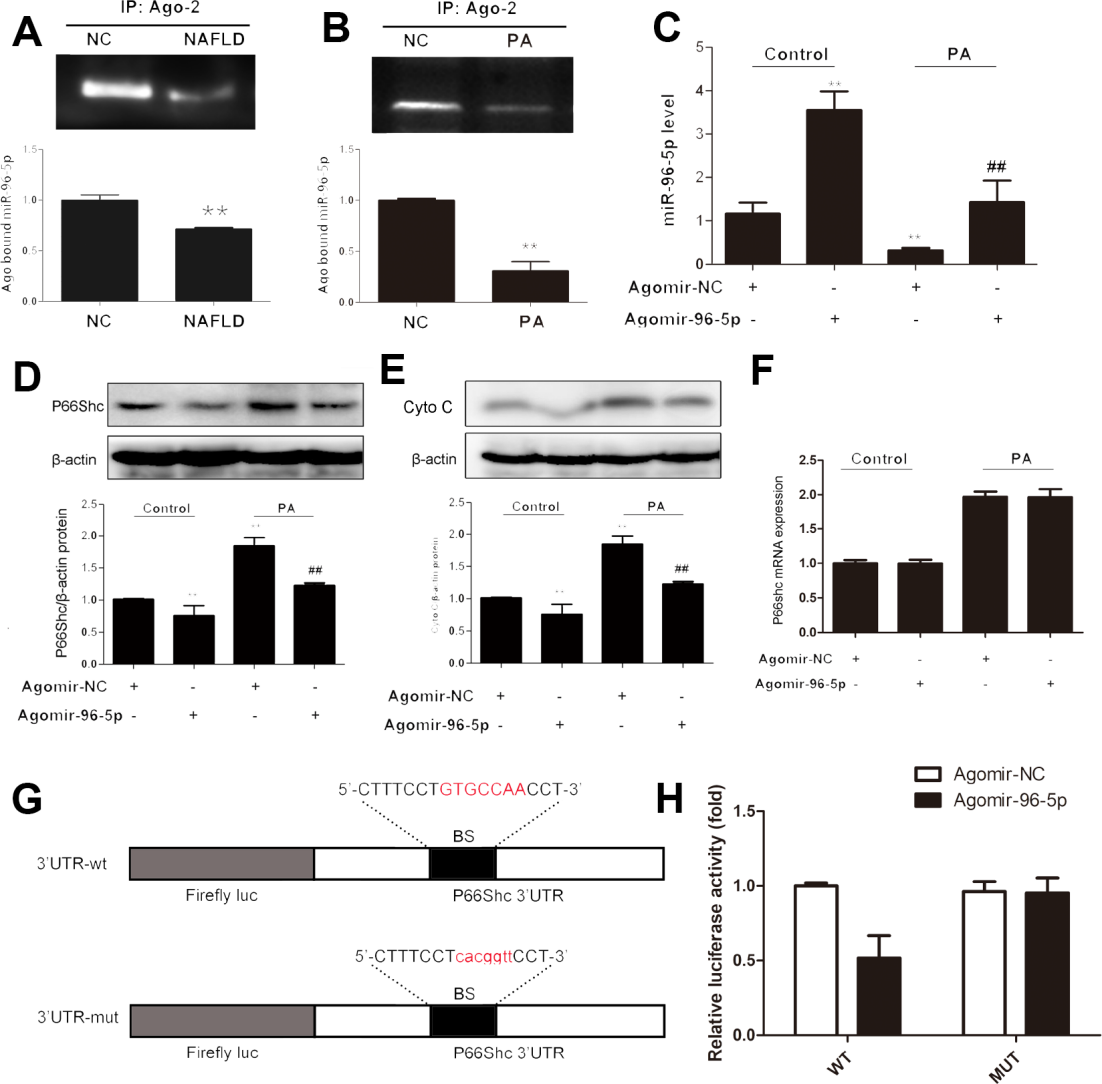
**miR-96-5p regulated p66shc expression in hepG2 cells.** HepG2 cells were transfected with ago-96-5p to upregulate miR-96-5p expression. Ago-NC was used as a normal control. (**A**, **B**) Argonaute-2 (Ago2) – immunoprecipitated miR-96-5p expression in NAFLD mice and PA-treated hepG2 cells. (**C**) miR-96-5p expression. (**D**, **E**) P66shc and cyto C protein expressions. (**F**) P66shc mRNA expression. (**G**) Schematic of the wild-type p66shc 3′UTR (3′UTR-wt) and mutated p66shc 3′UTR (3′UTR-mut) luciferase constructs. (**H**) HepG2 cells were transfected with 3′UTR-wt or 3UTR-mut and with ago-96-5p or ago-NC, as indicated. ^**^P< 0.05. Error bars depict the standard deviation. BS: binding site; NC: negative control.

To evaluate the potential roles of miR-96-5p in NAFLD, agomir targeting miR-96-5p was employed in the in vitro NAFLD model. MiRNA function requiring them to be uploaded in to the RISC, where targeting of miRNAs to mRNA occurs. Argonaute-2 was eluted from mice liver or PA-treated and miRNA was extracted. Levels of Ago-2-bound miR-96-5p were significantly decreased in NAFLD models both in vivo and in vitro compared with controls (Figure 6A, 6B). Agomir-96-5p treatment significantly increased miR-96-5p levels in the control and PA-treated cells compared with each agomir-NC (Figure 6C), p66shc protein expression was significantly down regulated following agomir-96-5p transfection as well as cytochrome C protein expression (Figure 6D, 6E). However, no apparent change in p66shc mRNA was observed in either group (Figure 6F). These data suggested that miR-96-5p had the potential to regulate hepatic p66shc expression, and this effect might involve repressing translation rather than affecting mRNA degradation.

To further validate whether p66shc was a direct target of miR-96-5p in the liver, luciferase fusion construct containing either the wild type or mutated p66shc 3′- UTR was transfected into hepG2 cells (Figure 6G). Then, these cells were con-transfected with agomir-96-5p and the corresponding control. As shown in Figure 6H, agomir-96-5p clearly suppressed the luciferase activity of the wild-type reporter. However, this repression was not observed for the mutated p66shc 3′-UTR. Collectively, these data indicated that miR-96-5p could indeed regulate p66shc expression in the liver.

### Catalpol alleviated ROS, apoptosis and hepatic steatosis might through upregulating miR-96-5p/p66shc pathway

**Figure 7:**
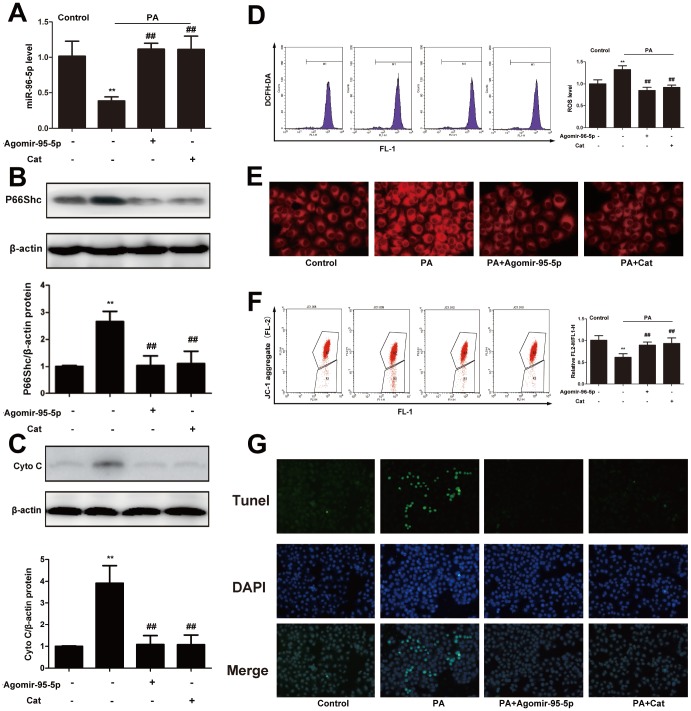
**Catalpol ameliorated oxidative stress, hepatic steatosis and apoptosis through upregulating miR-96-5p.** (**A**) miR-96-5p level. (**B**) P66Shc protein expression. (**C**) Cyto C protein expression. (**D**) ROS level. (**E**) Nile red staining. (**F**) JC-1 level. (**G**) Tunel staining. The results are the mean ± SD (n=8), ^**^P< 0.05 vs. Control group, ^##^P < 0.05 vs. WD group.

To better understand the role of miR-96-5p in ROS production and hepatic steatosis, agomir-96-5p was transfected into hepG2 cells, also, the effect of catalpol was evaluated comparing with agomir-96-5p. Agomir-96-5p and catalpol significantly increased miR-96-5p levels compared with PA-treated hepG2 cells (Figure 7A). To further explore the potential mechanisms, we examined the expression of p66shc. In PA-treated group, p66shc expression was markedly increased; however, agomir-96-5p or catalpol treatment enhanced miR-96-5p expression, and suppressed p66shc and cytochrome C protein expression effectively (Figure 7B, 7C). As expected, PA-induced ROS overproduction and hepatic steatosis were also attenuated by agomir-96-5p or catalpol (Figure 7D, 7E). Furthermore, agomir-95-5p or catalpol preserved mitochondrial membrane potential and reduced cell apoptosis (Figure 7F, 7G). Moreover, catalpol treatment significantly reversed the decreased miR-96-5p expression induced by PA. On the other hand, Antagomir-96-5p aggregated PA-induced hepatic steatosis and oxidative stress in hepG2 cells, moreover antagomir-96-5p blocked the inhibitory effects of catalpol on p66shc protein expression, hepatic steatosis and oxidative stress (Supplementary Figure 5). Taken together, the results indicated that catalpol performed such protective effects might through upregulate miR-96-5p expression level.

## DISCUSSION

NAFLD is the hepatic manifestation of metabolic syndrome. In recent years, the incidence of NAFLD was increasing. As currently available therapeutic approaches to NAFLD show rather limited effectiveness, novel treatment strategies are required. *Rehmannia glutinosa* has been widely used for thousands of years in traditional Chinese medicine with little reported toxicity, and catalpol is extracted from the root of *Rehmannia glutinosa*. Effects of catalpol on ameliorating NAFLD have been investigated by attenuating endoplasmic reticulum stress and NOX4 overexpression in our previous study, which indicating catalpol may produce such protective effects via inhibiting oxidative stress [19]. P66shc is a key modulator in oxidative stress. Thus, the present study represents attempted to demonstrate that 1) p66shc expression was significantly increased in NAFLD mice, 2) the p66shc/cytochrome C pathway was a pivotal therapeutic target for preventing NAFLD progression via inhibiting ROS overproduction, cell apoptosis and hepatic steatosis, 3) miR-96-5p could negatively regulate p66shc protein expression via directly binding to p66shc mRNA, 4) the protective effect of catalpol might be associated with upregulating miR-96-5p levels.

NAFLD is characterized by hepatic intracellular lipid accumulation. In the present study, furthermore, we found the role of catalpol ameliorating hepatic lipid accumulation and injury in HFD-, oxLDL- and PA-induced NAFLD. Catalpol decreased lipid levels such as TC, TG and FFA in the serum of HFD-induced mice. In addition, HE staining and transmission electron microscopy demonstrated a decrease of hepatic massive lipid accumulation in HFD-induced mice. Consistent with experiments in mice, Nile red staining showed that catalpol reduced the increase of lipid accumulation in oxLDL- and PA-treated hepG2 cells. Moreover, the decreased ALT and AST levels by catalpol in HFD-induced mice also showed an improvement on liver function. Therefore, catalpol has remarkable favorable functions for treating NAFLD.

Oxidative stress is an important feature of several liver diseases especially in HFD and saturated fatty acid such as PA induced NAFLD [20]. Previous studies demonstrated a critical role of oxidative stress in the pathogenesis of NAFLD [21, 22]. Excessive lipid accumulation increases oxidative stress and consequently develops into metabolic syndrome [23, 24]. Moreover, oxidative stress is mediated by multiple active species by different mechanisms and the same lipid oxidation products are produced by different active species involved [25]. Further, the effects of various antioxidants have been assessed in human and animal studies, but the underlying mechanism inhibiting oxidative stress during NAFLD remains in need of further investigation. In the present study, catalpol exhibited beneficial effects against oxidative stress in NAFLD by increasing GSH content and SOD activity but decreasing levels of MDA and LDH in serum of HFD-fed mice and in PA-induced HepG2 cells.

P66shc is an adaptor protein, which can play as an oxidoreductase that promotes aging in mammals by producing ROS in mitochondria. In response to several stimuli, p66Shc migrates into mitochondria where it catalyzes electron transfer from cytochrome C to oxygen resulting in hydrogen peroxide formation [26]. Deletion of p66Shc has been shown to reduce I/R injury as well as vascular abnormalities associated with diabetes and ageing. P66Shc-induced ROS formation is also involved in insulin signaling and might contribute to self-endogenous defenses against mild I/R injury. Recent findings raised possibility that p66shc is a contributing factor to the development of hepatic steatosis [7]. Raw data retrieved from ‘GSE94754’ in the GEO dataset showed a higher expression of P66Shc in the HFD-fed mice indicating an important role of P66Shc. In our NAFLD model, the upregulation of p66shc, which is a sign of oxidative stress, was found in livers of HFD-fed mice and in PA-induced HepG2 cells. Furthermore, ROS level was also increased in the livers of HFD-fed mice and in PA-induced HepG2 cells, suggesting that p66shc mediated pathway of oxidative stress might involve in NAFLD progress. Further, by using p66shc specific siRNA, we found that suppression of p66shc expression significantly blocked lipid accumulation and hepatic apoptosis in PA and oxLDL-induced HepG2 cells by Nile red staining and Tunel staining, indicating downregulation of p66shc expression benefits to ameliorate NAFLD. As shown in the results, high grade of p66shc protein expression and ROS accumulation in liver of HFD-fed mice and HepG2 cells induced by PA or oxLDL were significantly reversed by catalpol, indicating that catalpol may protect mice from NAFLD via a p66shc related pathway.

Hepatic apoptosis has been shown to be an important process of NAFLD and the mainly cause to induce hepatic oxidative injury. Cytochrome C is the hallmark of activation of the mitochondrial apoptotic pathway. As indicated in previous study, some lipid derivatives such as fat acids could directly inhibit several enzymes during the progress of NAFLD [27]. Moreover, saturated fat acids were able to active c-Jun N-terminal kinase and trigger the mitochondrial permeability transition, thus inducing mitochondrial release of cytochrome C and apoptosis. Previous studies also indicated increased expression of cytochrome C was found in both NAFLD and alcohol-induced liver apoptosis [9, 28]. In addition, a study on hepatic cancer cells reported that cytochrome C-induced apoptosis was mediated by ROS and Sirt1 related pathway [29]. Similarly, in our study, increases of cytochrome C protein expressions were detected in HFD-fed NAFLD mice, PA and oxLDL-treated hepG2 cells. Catalpol decreased cytochrome C protein expression, reversed the decreased mitochondrial membrane potential and reduced the number of apoptosis cells.

It is well known that increased p66shc expression is closely associated with oxidative stress and recent study reported that oxidative stress is related to cytochrome C-mediated apoptosis. Consistent evidence demonstrated that high expression of p66shc in ischemia reperfusion [26], renal tubular oxidative injury [30], angiotensin II-induced mouse hippocampal HT22 cells apoptosis [31] could active cytochrome C-mediated apoptotic pathways. However, the relationship between p66shc and cytochrome C in NAFLD haven’t been clearly demonstrated. In the present study, specific inhibit p66shc its siRNA blocked the increased cytochrome C protein expression and the following apoptosis, indicating p66shc-cytochrome C link is an important part in regulating hepatic apoptosis. These results demonstrated that the pathologic process in NAFLD is accompanied by changes in cytochrome C-mediated apoptotic signaling pathways. Further studies are needed to clarify how these signaling pathways regulate apoptosis in NAFLD.

Recent studies demonstrated the important roles of miRNAs in the pathogenesis of NAFLD, and regulation of several miRNAs, including miR-34a, miR-7a [32], miR-132 [33], and miR-378 [34], was previously shown to have direct or indirect protective effects on NAFLD. Plasma miR-17, miR-20a, miR-20b and miR-122 were also found to be potential biomarkers for diagnosis of NAFLD in type 2 diabetes mellitus patients [35]. Inhibition of miR-34a has been investigated to activate Sirt1/p66shc pathway to against rat NAFLD via inhibiting oxidative stress and hepatic apoptosis [6]. These results suggested that miRNAs played important roles in regulating oxidative stress and hepatic steatosis. However, whether miRNA was involved in the regulation of p66shc in NAFLD remains unclear. Several miRNAs have been shown to regulate p66shc expression in different disease models, including let-7 in the senescence of human diploid fibroblasts [36], miR-200c in endothelial cell injury [37], and miR-5582-5p in cancer cells proliferation [38]. Based on the prediction programs miRanda, TargetScan, and PicTar, miR-9-5p, miR-124-3p and miR-96-5p showed high potency to binding SHC1. Increased expression of miR-9-5p, miR-124-3p and decreased miR-96-5p level were detected. In the current view, miRNAs can negatively regulate gene expression by binding with the 3’-UTR of a specific mRNA, causing its degradation or translational repression. Thus, role of miR-96-5p was investigated in the current study.

Recent studies have revealed alterations in miR-96-5p expression in hepatocellular carcinoma [39], colorectal cancer [40], and pancreatic carcinoma [41]. In addition, miR-96-5p was considered to regulate glutathione levels [42]. Although alterations in miR-96-5p expression have been reported in certain types of cancer, its potential role in NAFLD and hepatic steatosis-induced apoptosis have not been demonstrated. Then, based on the above observations, we hypothesized the miR-96-5p might inhibit p66shc in a NAFLD model and thus affect oxidative stress and hepatic steatosis. As expected, decreased expression of miR-96-5p level was found in HFD-induced NAFLD mice model and PA-induced hepG2 cell models. Furthermore, downregulation of miR-96-5p was accompanied by increased p66shc levels. Combined with the results of the luciferase activity assays, the findings indicated that miR-96-5p had the potential to suppress p66shc in the liver, thereby affecting NAFLD. Also, catalpol treatment significantly increased miR-95-5p expression level, thus, we suggested that catalpol ameliorated NAFLD through upregulating miR-96-5p level.

**Figure 8:**
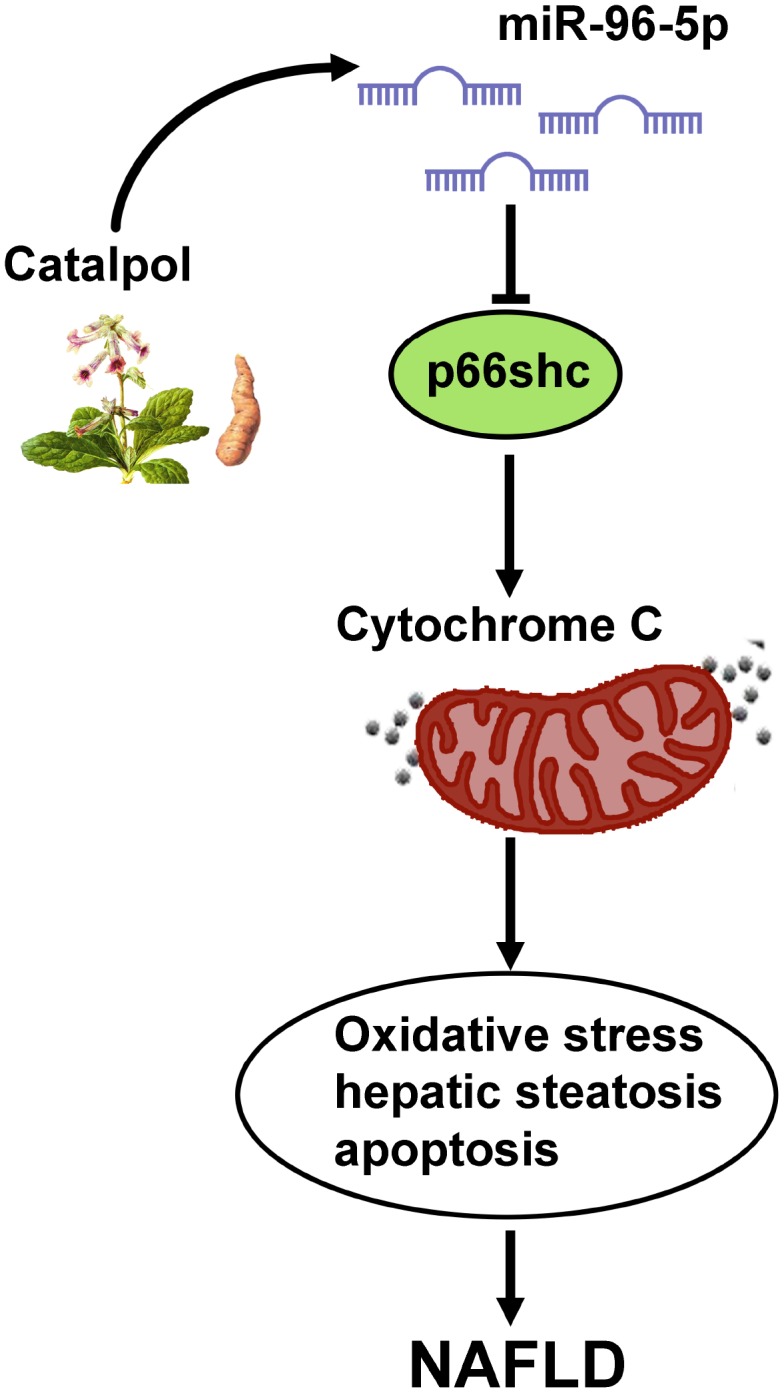
**Graphic summary for the mechanism that catalpol ameliorates NAFLD through upregulating miR-96-5p level via p66shc/cytochrome C cascade.**

In conclusion, as shown in Figure 8, we demonstrated that activation of p66shc/cytochrome C cascade was responsible to cause oxidative stress, hepatic steatosis and apoptosis in NAFLD. However, miR-96-5p was able to suppress p66shc/cytochrome C cascade via targeting p66shc mRNA 3’UTR and catalpol could lead to suppression of NAFLD via upregulating miR-96-5p level.

## MATERIALS AND METHODS

### Gene expression profile data

Data of GEO series GSE94754 was obtained from Gene Expression Omnibus database. The mice used for GSE94754 were exposed to control or high-fat diet for 12 weeks. Samples for microarrays analysis were gained from the liver tissue, with 8 samples of high fat diet and 8 samples of normal diet as control group.

### Raw data preprocessing and screening and integration of differentially expressed genes

R_4.4 was used to screen the differentially expressed genes between two different diet groups. Genes integration of differentially expressed genes identified from gene chips was performed using RoubustRank Aggreg. Genes with statistic significance (p<0.05, fold change>2) were filtered. Heat maps were generated by ‘pheatmap’ package using data with expression.

### Reagents

Catalpol (98%) was obtained from Nanjing jingzhu Biotech Ltd. Co (Nanjing, China). 1640 medium was bought from Gibco-BRL Company (Gaithersburg, MD, USA). DCFH-DA fluorescent probe and ECL Plus were obtained from Beyotime (Jiangsu, China). Antibodies specific for P66Shc, cytochrome C was obtained from Proteintech Group (Wuhan, China). Antibody specific for β-actin was purchased from Beyotime (Jiangsu, China).

### Ethics statement

All experiments were approved by the Animal Care and Use Committee of Dalian Medical University, and the experimental procedures were performed in strict accordance with Legislation Regarding the Use and Care of Laboratory Animals of China. Before the experiments, the animals were allowed to suit the new environment for 7 days, and housed in a room under 12 h light/dark cycle, a controlled temperature at 22± 3 °Cand a relative humidity at 60± 10%.

### Animals and diets

Male eight-week-old LDL receptor knockout (LDLr-/-) mice with a C57BL/6 background were purchased from Vital River Laboratory Animal Technology Co., Ltd. (Beijing, China). To detect the effect of catalpol on atherosclerosis, sixty LDLr-/- mice were divided into five groups: chow diet (n=12), catalpol (100mg/kg/D, n=12), western diet (HFD, n=12), western diet with catalpol (100mg/kg/D, n=12) and western diet with catalpol (200mg/kg/D, n=12) for 16 weeks. The diet was a commercially prepared mouse food (MD12017) supplemented with 20.0% (wt/wt) cocofat,1.25% (wt/wt) cholesterol, and 22.5% (wt/wt) protein and 45.0% carbohydrate (Jiangsu Medicine Ltd., Jiangsu,, China). At week-16, mice were anesthetized with 2% isoflurane (Forene®, Abbott), one-milliliter of blood was collected by abdominal aorta, and tissues were collected for further analysis.

### Biochemical analyses

Blood samples were centrifuged (3000 rpm, 10 min, 4 C) and serum was separated for analysis. Total cholesterol (TC), triglyceride (TG), alanine aminotransferase (ALT), aspartate aminotransferase (AST), superoxide dismutase (SOD), malondialdehyde (MDA), catalase (CAT), ATP content and ATPase activity levels of serum were measured using commercial kits purchased from Nanjing Jiancheng Bioengineering Institute (Nanjing, China). All kits were used according to the corresponding manufacturers' instructions.

### Liver histological analysis

For pathological analysis, the isolated left lateral segment of the liver lobes was fixed into 4% paraformaldehyde solution. Then the fixed-livers were embedded in paraffin and sliced (5 μM sections). Finally, the sections were stained with hematoxylin and eosin for histologic analysis.

### Electron microscopy

For ultrastructural analysis by electron microscopy, the liver samples of control, high fat diet (HFD) and high-dose catalpol treatment group were fixed in 2.5% glutaraldehyde solution and embedded according to standard protocols. Briefly, the samples were rinsed in PBS and then postfixed in 1% osmium tetroxide. After rinsing in PBS for 45 min, the samples were dehydrated in a graded series of ethanol (70–100%) and then routinely embedded in epon. Then, embedded specimens were cut into ultrathin sections of 0.5 μM. Finally, ultrathin sections were stained with uranyl acetate and lead citrate.

### Preparation of ox-LDL

Fresh whole blood from normal human was added with 0.5% EDTA-2Na for anticoagulation. Plasma was isolated by spinning the blood at 4°C, 7000r/min for 15 min. Native LDL (density: 1.019~1.063 g/mL) was separated from the fresh nomolipidemic human serum by discontinuous density-gradient ultracentrifugation using a Beckman coulter optima L-100 XP Ultracentrifuge and then oxidatively modified [43]. LDL was oxidized with 50 μM CuSO_4_ at 37°C for 24 h, and then transferred into EDTA (200 mmol/L) in PBS for 24 h at 4°C. Subsequently, oxidation was stopped by extensive dialysis against PBS with 0.01% EDTA and sterilized by filtration. LDL oxidation was confirmed by thiobarbituric acid reaction substances with malondialdehyde as the standard [44]. LDL and oxLDL protein concentrations were determined with a bicinchoninic acid (BCA) protein assay kit (Beyotime) which used bovine serum albumin as the standard and was expressed as micrograms per milliliter of solution. LDL prepared every 2 weeks.

### Cell culture and treatment

Human hepatocellular carcinoma cell line HepG2 cells were purchased from ScienCell company (CA, USA) and cultured in Dulbecco's Modied Eagle's Medium (DMEM) with 10% (v/v) fetal bovine serum (Gibco, CA, USA), 20 mg/mL penicillin, and 20 mg/mL streptomycin and maintained at 37°C in a humidified atmosphere of 5% CO_2_. One day before treatment, the culture medium was changed to DMEM medium without FBS. Palmitate (PA) was dissolved in PBS at 70°C and then mixed with 10% BSA at 55°C for 10 min to achieve a final PA concentration of 500 μM. The experiments were divided into six groups: control; induction with 20 μM catalpol; hepatic steatosis induction with 500 μM PA or ox-LDL (200μg/mL); concomitant induction with 5, 20, 80μM catalpol and 500 μM PA or ox-LDL (200μg/mL). Briefly, HepG2 cells were seeded on 6-well plates at a density of 1×10^6^ per well and allowed to grow to desired confluence. Then the cells were treated with indicated concentrations of PA or ox-LDL and catalpol for 24 hours and the cells or culture media were collected to analysis.

### Transfection of agomirs/antagomirs and siRNA

HepG2 cells were transiently transfected using Lipofectamine 2000 (Life Technologies-Invitrogen, Carlsbad, CA, USA) in Opti MEM according to the manufacturer’s protocol, as described [45]. Agomirs/antagomirs, siRNA targeting p66shc (siRNA) and the corresponding NC were obtained from GenePharma. The transfection procedures were performed according to the manufacturer’s protocols.

### Measurement of intracellular ROS

2’, 7’-dichlorodihydrofluorescein diacetate (H_2_DCFDA) probe was employed to measure ROS level as previously described [46]. HepG2 cells were incubated with PA and different concentrations of catalpol for 24h at 37°C and then collected and incubated with 20 μM H_2_DCFHDA for 30min at 37°C. The fluorescence intensity was immediately measured using Fluorescent Activated Cell Sorting (FACS) Calibur Flow Cytometer (Becton Dickinson Immunocytometry Systems, CA, USA) equipped with an argon ion laser (488nm excitation) and 20 000 cells per sample were measured.

### Mitochondria membrane potential assay

The mitochondrial membrane potential was assessed in the parasite using JC-1 (5, 5’, 6, 6’-tetrachloro-1, 1′, 3, 3′- tetraethylbenzimidazolylcarbocyanine iodide), that remains in monomeric form in the cytoplasm and has a green fluorescence (525 nm). However, the membrane potential of functional mitochondria establishes a negative charge that allows the lipophilic dye to accumulate and form aggregates in the mitochondria, which have red fluorescence (590 nm). HepG2 were collected from parasite cultures in control and experimental sets at different time points and incubated with JC-1 dye (at a final concentration of 10 μM) for 30 min at 37°C. Cells were washed with PBS and analyzed by flow cytometry using FACSCalibur flow cytometer and CellQuestPro software (Becton Dickinson, San Jose, CA, USA).

### Nile red staining

Nile red was prepared as previously described [47]. The lipid-bound Nile Red fluorescence was observed with a fluorescence microscope.

### Argonaute-2 (Ago-2) immunoprecipitation

Pools of three individual liver for each group were homogenized in immunoprecipitation buffer (300 mM NaCl, 5 mM MgCl2, 0.1% NP-40, 50 mM Tris–HCl, pH7.5), centrifuged, and 400 μL lysate was incubated overnight at 4°C with 5 μg of antibodies to Argonaute-2 (Cell Signaling Technology, Beverly, MA, USA). 20 μL of 50% Protein-A/G-agarose beads (Beyotime Institute of Biotechnology) was then added for 1h at 4°C and the bead complexes were centrifuged at 16,000 ×g for 15 min at 4°C. The pellet was washed and processed for miRNA extraction, stem-loop RT, and RT–PCR (Applied Biosystems).

### Western blot analysis

Cells and tissues were harvested and protein extracts prepared according to established methods [48]. Extracts were separated in sodium dodecyl sulfate–polyacrylamide electrophoresis gels (8~15%) and transferred to a polyvinylidene difluoride (PVDF) membrane (Millipore, Bedford, MA, USA). The membranes were blocked with 5% milk, and then incubated with indicated primary antibodies at 4°C overnight. After washing, the membranes were then incubated with the appropriate secondary antibodies. The membranes were exposed to enhance chemiluminescence-plus reagents (Beyotime Institute of Biotechnology, Hangzhou, China). The emitted light was captured by a Bio-rad imageing system with a Chemi HR camera 410 and analyzed with a Gel- Pro Analyzer Version 4.0 (Media Cybernetics, MD, USA).

### miRNA expression

Total RNA was extracted using the miRNeasy kit (Qiagen Shanghai, China) and 250 ng was reverse transcribed using stem-loop Multiplex primer pools (Applied Biosystems, Foster City, CA, U.S.A.). Reverse transcription (RT)–specific primers for rat miRNAs miR-9-5p, miR-96-5p, miR-124-3p (life technology) were used for all miRNA RT. Quantitative polymerase chain reaction(qPCR) was carried out using a 7500HT Fast Realtime System (Applied Biosystems) and TaqMan microRNA assays (Applied Biosystems). Endogenous RNA U6 small nuclear 2 (RNU6B) was used for normalization. The relative fold change in expression of the target gene transcript was determined using the comparative cycle threshold method (2^-ΔΔCT^).

### TUNEL staining

Apoptosis was quantified in paraffin-embedded liver using a terminal deoxynucleotidyl transferase-mediated deoxyuridine triphosphate nick-end labelling (TUNEL) Assay kit (TUNEL Apo-Green Detection Kit, Biotool, Houston, USA)) according to manufacturer instructions. Green fluorescence staining indicated positive TUNEL staining.

### Luciferase activity assay

Plasmids containing the SHC1 3′-UTR response element (3’UTR-wt) and the corresponding mutant (3’UTR-mut) were obtained from Obio Technology Corp., Ltd (Shanghai, China). Plasmid DNA and the agomir or the NC was conducted 36 h after transfection. The luciferase activity was determined with a Dual-Luciferase Reporter Assay Kit (TransGen) using a Dual-Light Chemiluminescent Reporter Gene Assay System (Berthold) and was normalized to the Renilla luciferase activity.

### Measurement of 8OH-dG contents

DNA was isolated according to the protocol’s instruction, and the RNA-free DNA obtained was used to determine 8-OHdG levels using the 8-OHdG ELISA kit (Huamei, Wuhan, China) according to the manufacturer’s instructions.

### Statistical analysis

All results are expressed as the mean± standard deviation (SD) from more than three independent experiments, and data analyses were performed with the SPSS software package, version 19.0. Comparison of quantitative variables was performed by either Student’s t test or ANOVA followed by the student-newman-keuls (SNK) test. p values <0.05 (two-tailed) were considered statistically significant.

## Supplementary Material

Supplementary Figures
