# Supplementary material for: Targeting of miR-96-5p by catalpol ameliorates oxidative stress and hepatic steatosis in LDLr-/- mice via p66shc/cytochrome C cascade
Source: Aging (Albany NY). 2020 Feb 5;12(3):2049–69. doi: 10.18632/aging.102721 (PMC7041734; doi:10.18632/aging.102721)
Supplement: Supplementary Figures [file aging-12-102721-s001..pdf]

SUPPLEMENTARY FIGURES

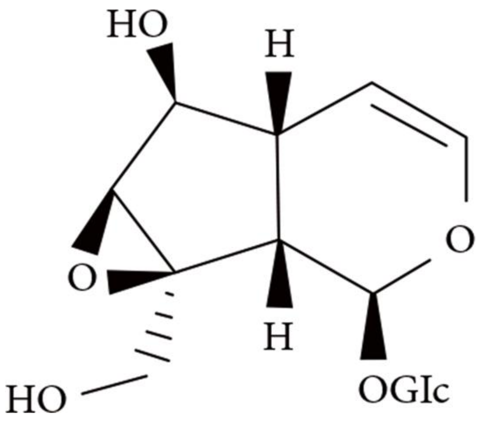

Supplementary Figure 1. Chemical structure of catalpol.

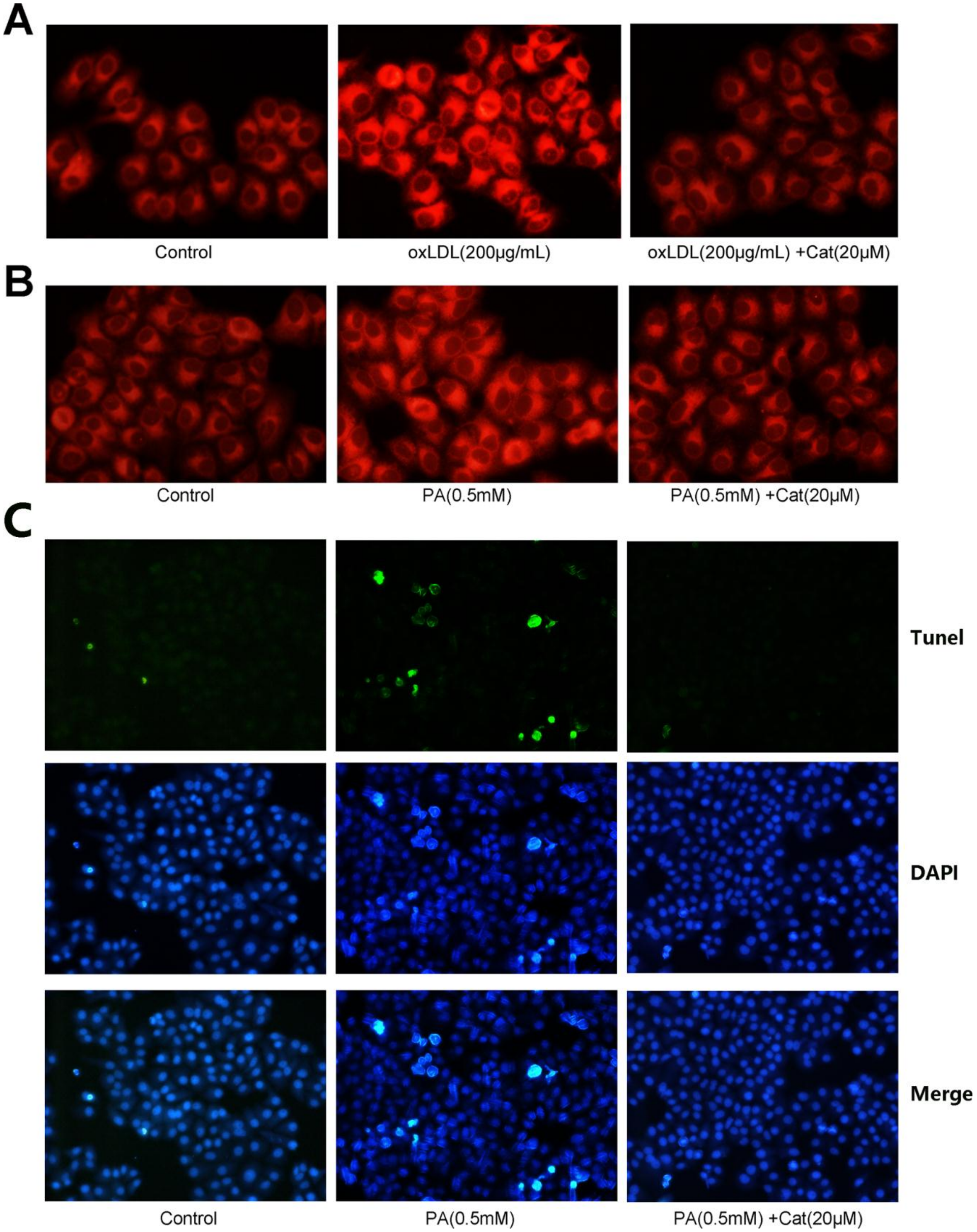

Supplementary Figure 2. Catalpol inhibited hepatic steatosis and cell apoptosis in hepG2 cells. (**A**) Catalpol inhibited hepatic steatosis in oxLDL-treated hepG2 cells. (**B**) Catalpol inhibited hepatic steatosis in PA-treated hepG2 cells. (**C**) Catalpol inhibited apoptosis in PA-treated hepG2 cells.

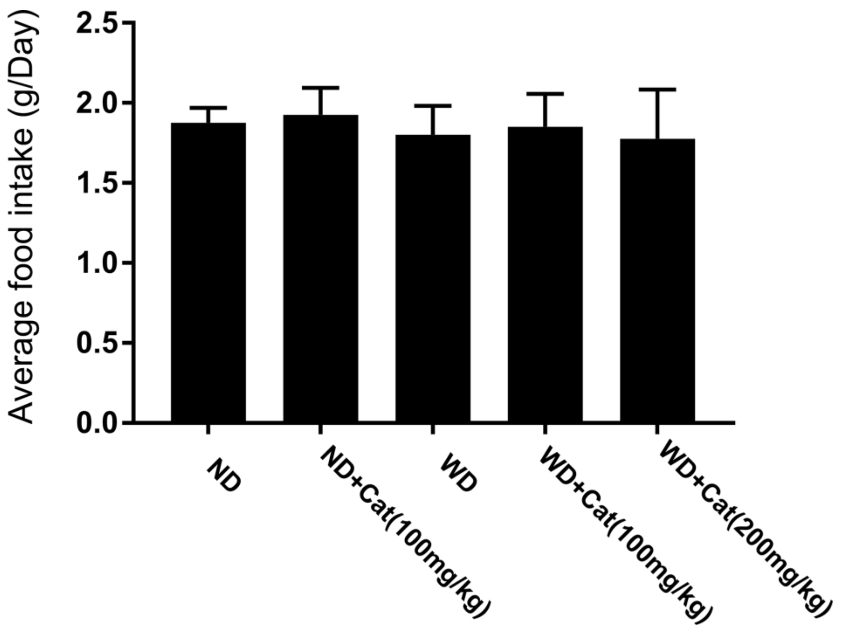

Supplementary Figure 3. Food intake of the mice. The results are the mean± SD (n=8).

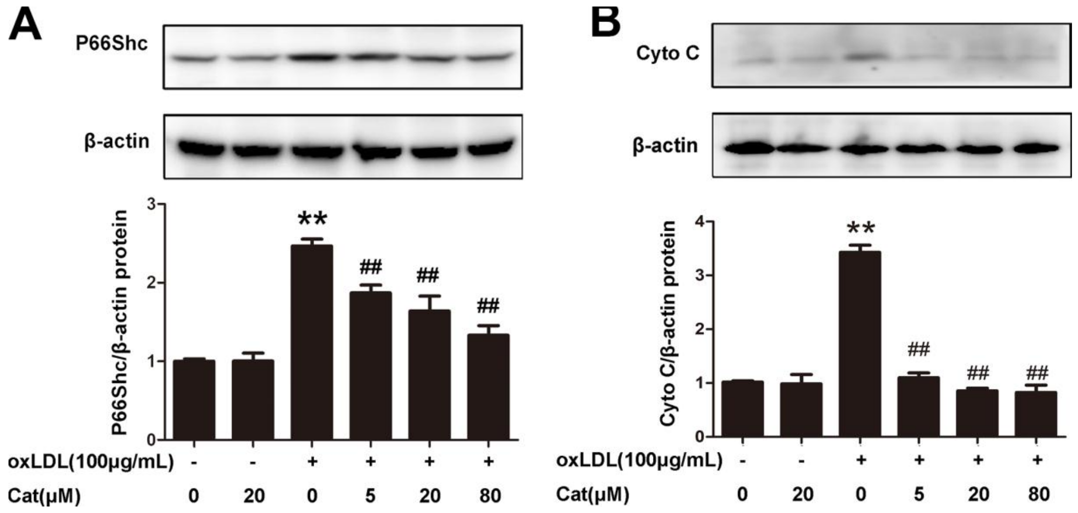

Supplementary Figure 4. P66shc and cyto C protein expressions in oxLDL-treated hepG2 cells. (**A**) P66shc protein expression. (**B**) cyto C protein expression. The results are the mean± SD (n=8), $^{**}P < 0.05$ vs. Control group, $^{\#\#}P < 0.05$ vs. oxLDL group.

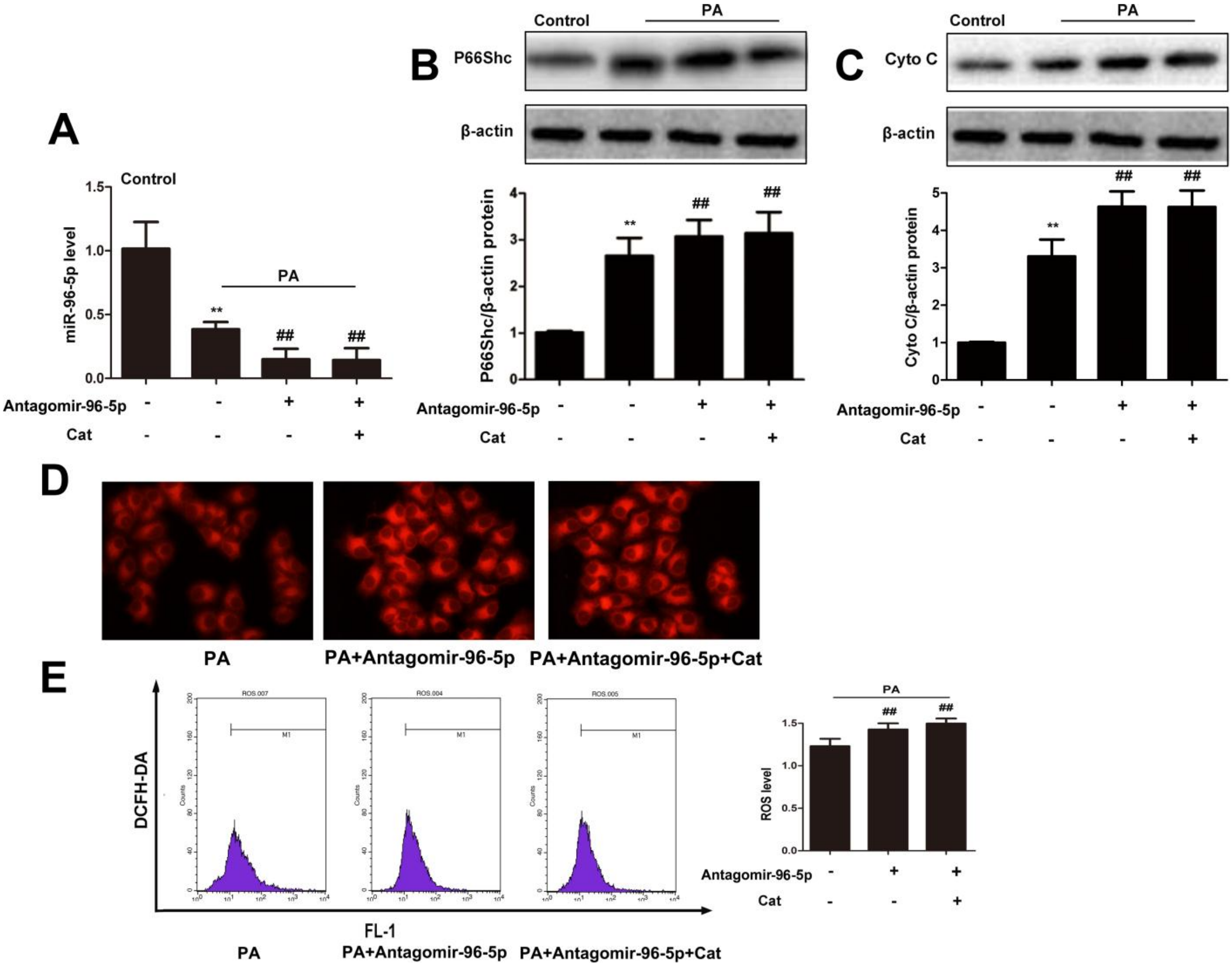

Supplementary Figure 5. Antagomir-96-5p aggregated PA-induced hepatic steatosis and oxidative stress. (**A**) miR-96-5p level. (**B**) P66Shc protein expression. (**C**) Cyto C protein expression. (D) Nile red staining. (**E**) ROS level. The results are the mean± SD (n=8), **P< 0.05 vs. Control group, $^{\#\#}$P < 0.05 vs. PA group.
